# A research agenda to improve incidence and outcomes of assisted vaginal birth

**DOI:** 10.2471/BLT.23.290140

**Published:** 2023-10-04

**Authors:** Ana Pilar Betrán, Maria Regina Torloni, Fernando Althabe, Elena Altieri, Sabaratnam Arulkumaran, Fatema Ashraf, Patricia Bailey, Mercedes Bonet, Maurice Bucagu, Emma Clark, Nasrin Changizi, Robyn Churchill, Sunday Dominico, Soo Downe, Tim Draycott, Arfang Faye, Claire Feeley, Diederike Geelhoed, Atf Gherissi, Karima Gholbzouri, Gagan Grupta, Tedbabe Degefie Hailegebriel, Claudia Hanson, Katharina Hartmann, Lubna Hassan, George Justus Hofmeyr, Anoma Chandani Jayathilaka, Charles Kabore, Nancy Kidula, Carol Kingdon, Oleg Kuzmenko, Pisake Lumbiganon, Glen DL Mola, Allisyn Moran, Bremen de Muncio, Barbara Nolens, Newton Opiyo, Robert C Pattinson, Mariana Romero, Jos van Roosmalen, Monica M Siaulys, Jose Simon Camelo, Jeffrey Smith, Howard L Sobel, Soha Sobhy, Claudio Sosa, Joao Paulo Souza, Petra ten Hoope-Bender, Shakila Thangaratinam, John Varallo, Alison Wright, Ann Yates, Olufemi O Oladapo

**Affiliations:** a Department of Sexual and Reproductive Health and Research, World Health Organization, Avenue Appia 20, 1202 Geneva, Switzerland.; b EBH Postgraduate Programme, Department of Medicine, Sao Paulo Federal University-UNIFESP, Sao Paulo, Brazil; c Buenos Aires, Argentina; d Behavioural Insights Unit, Department of Communications, World Health Organization, Geneva, Switzerland; e St George's University of London, London, United Kingdom of Great Britain and Northern Ireland; f Department of Obstetrics & Gynaecology, Shaheed Suhrawardy Medical College Hospital, Dhaka, Bangladesh; g Reproductive, Maternal, Newborn and Child Health, FHI 360, Durham, North Carolina, United States of America; h Department of Maternal, Newborn, Child and Adolescent Health and Ageing, World Health Organization, Geneva, Switzerland; i Maternal Child Health and Nutrition, USAID Bureau for Global Health, Arlington, United States; j Maternal, Fetal and Neonatal Research Center, Tehran University of Medical Sciences, Tehran, Iran; k Thamini Uhai (Value Life), United Republic of Tanzania; l Department of Midwifery, University of Central Lancashire, Preston, United Kingdom; m The Chilterns, Southmead Hospital, Bristol, United Kingdom; n Reproductive, Maternal, Newborn, Child and Adolescent Health Unit, Ministry of Health, Gambia; o School of Community Health and Midwifery, University of Central Lancashire, Preston, United Kingdom; p Tete, Mozambique; q High School for Health Science and Techniques, University of Tunis El Manar, Tunis, Tunisia; r WHO Regional Office for the Eastern Mediterranean, Cairo, Egypt; s United Nations Children's Fund, New York, United States; t Department of Global Public Health, Karolinska Institute, Stockholm, Sweden; u Mother Hood e.V. - Federal Parents' Initiative for the Protection of Mother and Child during Pregnancy, Bonn, Germany; v Women's Health Intervention and Development Initiative, Islamabad, Pakistan; w Department of Obstetrics and Gyneacology, University of Botswana, Gaborone, Botswana; x WHO Regional Office for South-East Asia, New Delhi, India; y Institut de Recherche en Sciences de la Sante, Ouagadougou, Burkina Faso; z WHO Regional Office for Africa, Brazzaville, Congo; aa Research in Childbirth and Health Unit, University of Central Lancashire, Preston, United Kingdom; ab WHO Regional Office for Europe, Copenhagen, Denmark; ac Faculty of Medicine, Khon Kaen University, Khon Kaen, Thailand; ad School of Medicine and Health Sciences, University of Papua New Guinea; Port Moresby, Papua New Guinea; ae WHO Regional Office for the Americas, Montevideo, Uruguay; af Department of Obstetrics and Gyneacology, Canisius-Wilhelmina Hospital, Nijmegen, Kingdom of the Netherlands; ag Maternal and Infant Health Care Strategies unit, University of Pretoria, Pretoria, South Africa; ah Centro de Estudios de Estado y Sociedad Consejo Nacional de Investigaciones Científicas y Tecnológicas (CEDES), Buenos Aires, Argentina; ai Working Party for International Safe Motherhood and Reproductive Health, Groningen, Kingdom of the Netherlands; aj Hospital e Maternidade Santa Joana, Sao Paulo, Brazil; ak Riberao Preto Medical School, University of Sao Paulo, Sao Paulo, Brazil; al Maternal, Newborn & Child Health, Bill & Melinda Gates Foundation, Seattle, Washington, United States; am WHO Regional Office for Western Pacific, Manila, Philippines; an Women's Health Research Unit, Barts and The London School of Medicine and Dentistry, Queen Mary University of London, London, United Kingdom; ao Centro Hospitalario Pereira Rossel, Montevideo, Uruguay; ap Sao Paulo, Brazil; aq United Nations Population Fund, Geneva, Switzerland; ar Institute of Metabolism and Systems Research, University of Birmingham, Birmingham, United Kingdom; as Jhpiego, Baltimore, Maryland, United States; at International Federation of Gynaecologists and Obstetricians (FIGO), London, United Kingdom; au International Confederation of Midwives (ICM), The Hague, Kingdom of the Netherlands

## Abstract

Access to emergency obstetric care, including assisted vaginal birth and caesarean birth, is crucial for improving maternal and childbirth outcomes. However, although the proportion of births by caesarean section has increased during the last few decades, the use of assisted vaginal birth has declined. This is particularly the case in low- and middle-income countries, despite an assisted vaginal birth often being less risky than caesarean birth. We therefore conducted a three-step process to identify a research agenda necessary to increase the use of, or reintroduce, assisted vaginal birth: after conducting an evidence synthesis, which informed a consultation with technical experts who proposed an initial research agenda, we sought and incorporated the views of women’s representatives of this agenda. This process has allowed us to identify a comprehensive research agenda, with topics categorized as: (i) the need to understand women’s perceptions of assisted vaginal birth, and provide appropriate and reliable information; (ii) the importance of training health-care providers in clinical skills but also in respectful care, effective communication, shared decision-making and informed consent; and (iii) the barriers to and facilitators of implementation and sustainability. From women’s feedback, we learned of the urgent need to recognize labour, childbirth and postpartum experiences as inherently physiological and dignified human processes, in which interventions should only be implemented if necessary. The promotion and/or reintroduction of assisted vaginal birth in low-resource settings requires governments, policy-makers and hospital administrators to support skilled health-care providers who can, in turn, respectfully support women in labour and childbirth.

## Résumé

L'accès aux soins obstétriques d'urgence, y compris l'accouchement vaginal assisté et la césarienne, est essentiel pour améliorer les effets de la maternité et de l'accouchement. Toutefois, bien que la proportion de césariennes ait augmenté ces dernières décennies, le recours à l'accouchement vaginal assisté a diminué. C'est particulièrement le cas dans les pays à revenu faible ou intermédiaire, bien que l'accouchement vaginal assisté soit souvent moins risqué qu'une césarienne. Nous avons donc mené un processus en trois étapes afin d'imaginer un programme de recherche qui permettrait d'augmenter le recours à l'accouchement vaginal assisté ou de le réintroduire. Après avoir réalisé une synthèse des données probantes, qui a servi de base à une consultation avec des experts techniques qui ont proposé un programme de recherche initial, nous avons sollicité et incorporé les avis des représentantes des femmes pour ce programme. Ce processus nous a permis d'imaginer un programme de recherche complet, avec des sujets classés comme suit: (i) la nécessité de comprendre la perception qu'ont les femmes de l'accouchement vaginal assisté et de fournir des informations appropriées et fiables; (ii) l'importance de la formation des prestataires de soins de santé en matière de compétences cliniques, mais aussi de respect dans les soins de santé, de communication efficace, de prise de décision partagée et de consentement éclairé; ou (iii) les obstacles à la mise en œuvre et à la durabilité et les facteurs qui les facilitent. Les réactions de femmes nous ont appris qu'il était urgent de reconnaître que l'accouchement, la naissance et le post-partum sont des processus humains intrinsèquement physiologiques et dignes au cours desquels les interventions ne devraient être mises en œuvre qu'en cas de nécessité. La promotion et/ou la réintroduction de l'accouchement vaginal assisté dans les régions à faibles ressources nécessitent que les pouvoirs publics, les décideurs politiques et les administrations d'hôpitaux soutiennent les prestataires de soins de santé qualifiés, qui pourront à leur tour soutenir respectueusement les femmes pendant l'accouchement.

## Resumen

El acceso a la atención obstétrica de emergencia, incluido el parto vaginal asistido y el parto por cesárea, es crucial para mejorar los resultados de la maternidad y el parto. No obstante, aunque el porcentaje de partos por cesárea ha aumentado en las últimas décadas, el uso del parto vaginal asistido ha disminuido. Esto ocurre especialmente en los países de ingresos bajos y medios, a pesar de que un parto vaginal asistido suele ser menos arriesgado que un parto por cesárea. Por lo tanto, llevamos a cabo un proceso de tres pasos para identificar un programa de investigación necesario para aumentar el uso del parto vaginal asistido o volver a incorporarlo: tras realizar una síntesis de la evidencia, que sirvió de base para una consulta con expertos técnicos que propusieron un programa de investigación inicial, buscamos e integramos las opiniones de las representantes de las mujeres sobre este programa. Este proceso nos ha permitido identificar un programa de investigación exhaustivo, con temas categorizados como: (i) la necesidad de comprender las percepciones de las mujeres sobre el parto vaginal asistido, y proporcionar información adecuada y fiable; (ii) la importancia de formar a los profesionales sanitarios en habilidades clínicas, pero también en atención respetuosa, comunicación efectiva, toma de decisiones compartida y consentimiento informado; o (iii) las barreras y los facilitadores de la implementación y la sostenibilidad. A partir de las opiniones de las mujeres, nos enteramos de la urgente necesidad de reconocer las experiencias del parto, el alumbramiento y el posparto como procesos humanos inherentemente fisiológicos y dignos, en los que las intervenciones solo deben aplicarse si son necesarias. La promoción o la reincoporación del parto vaginal asistido en regiones de escasos recursos exige que los gobiernos, los responsables de formular políticas y los administradores de hospitales apoyen a los profesionales sanitarios capacitados que, a su vez, pueden ayudar a las mujeres en el trabajo de parto y el alumbramiento de manera respetuosa.

## ملخص

إن الوصول إلى رعاية التوليد الطارئة، بما في ذلك الولادة الطبيعية المدعومة والولادة القيصرية، هو أمر بالغ الأهمية لتحسين نتائج رعاية الأمومة والولادة. ومع ذلك، فعلى الرغم من زيادة نسبة الولادة القيصرية خلال العقود القليلة الماضية، فقد انخفض اللجوء إلى الولادة الطبيعية المدعومة. وهذا هو الحال بشكل خاص في الدول ذات الدخل المنخفض، والدول ذات الدخل المتوسط، على الرغم من أن الولادة الطبيعية المدعومة غالبًا ما تكون أقل خطورة من الولادة القيصرية. وعلى ذلك، فقد قمنا بإجراء عملية من ثلاث خطوات لتحديد أجندة البحث الضرورية لزيادة اللجوء إلى، أو إعادة طرح، الولادة الطبيعية المدعومة: بعد إجراء تجميع للأدلة، والذي كان بمثابة مشورة للخبراء الفنيين الذين اقترحوا أجندة بحث أولية، فقد سعينا وراء آراء ممثلي النشاء في هذه الأجندة، وقمنا بتضمين هذه الآراء فيها. وقد أتاحت لنا هذه العملية تحديد أجندة بحثية شاملة، مع تصنيف المواضيع كما يلي: (1) الحاجة إلى فهم تصورات النساء للولادة الطبيعية المدعومة، وتوفير معلومات مناسبة وموثوقة؛ أو (2) أهمية تدريب مقدمي الرعاية الصحية على المهارات الإكلينيكية، وكذلك على الرعاية المحترمة، والتواصل الفعال، وصنع القرار المشترك، والموافقة المستنيرة؛ أو (3) العوائق التي تواجه التنفيذ والاستدامة والعوامل المساعدة لهما. ومن خلال تعليقات النساء، تعلمنا الحاجة الملحة للاعتراف بتجارب المخاض، والولادة، وما بعد الولادة، باعتبارها عمليات إنسانية فسيولوجية وراقية بالفطرة، والتي لا ينبغي التدخل فيها، إلا إذا لزم الأمر. إن الترويج للولادة الطبيعية المدعومة، أو إعادة طرحها، في البيئات ذات الموارد المنخفضة، يتطلب من الحكومات، وواضعي السياسات، ومديري المستشفيات، دعم مقدمي الرعاية الصحية من ذوي المهارة، والذين يمكنهم بدورهم دعم النساء في المخاض والولادة بشكل محترم.

## 摘要

能够获得产科急诊护理（包括阴道助产分娩和剖腹产）对于改善孕产妇分娩结果至关重要。然而，尽管在过去几十年，剖腹产的比例有所增长，但使用阴道助产分娩的比例却有所下降。在低收入和中等收入国家尤其如此，尽管阴道助产分娩的风险往往低于剖腹产。因此，我们通过一个三步流程，确定了增加使用或重新引入阴道助产分娩所需的研究议程：我们在综合证据之后，咨询了提出初步研究议程的技术专家，并寻求和纳入了该议程中妇女代表的意见。这一过程使我们能够确定一个全面的研究议程，其主题分为：(i) 需要了解妇女对阴道助产分娩的看法，并提供适当和可靠的信息；(ii) 对医疗保健提供者进行临床技能培训的重要性，但也包括在尊重护理、有效沟通、共同决策和知情同意方面的培训；或 (iii) 在实施和可持续性方面的障碍和促进因素。从妇女的反馈中，我们了解到，现在迫切需要认识到分娩、生产和产后经历是与生俱来的生理过程和有尊严的人类过程，只有在必要时才应实施干预措施。在资源匮乏的环境中促进和/或重新引入阴道助产分娩需要政府、决策者和医院管理人员支持熟练的医疗保健提供者，这些提供者因此会尊重和支持分娩妇女。

## Резюме

Доступ к неотложной акушерской помощи, включая ассистированные вагинальные роды и кесарево сечение, имеет решающее значение для улучшения показателей исходов родов для матери и плода. Однако, несмотря на то что доля родов путем кесарева сечения за последние несколько десятилетий увеличилась, использование ассистированных вагинальных родов снизилось. Это особенно актуально для стран с низким и средним уровнем дохода, несмотря на то что ассистированные вагинальные роды часто менее рискованны по сравнению с кесаревым сечением. В связи с этим был проведен трехэтапный процесс определения программы исследований, необходимых для расширения использования или возобновления практики ассистированных вагинальных родов: после проведения синтеза доказательств, на основе которого проводились консультации с техническими специалистами, предложившими первоначальную программу исследований, были запрошены и учтены мнения представителей женщин относительно этой программы. Благодаря данному процессу была разработана комплексная программа исследований, в которую вошли такие темы, как: (i) необходимость понимания того, как женщины воспринимают ассистированные вагинальные роды, и предоставления соответствующей и достоверной информации; (ii) важность обучения медицинских работников не только клиническим навыкам, но и уважительному подходу в лечении, эффективному общению, совместному принятию решений и информированному согласию; (iii) барьеры и факторы, способствующие внедрению и устойчивому развитию. На основании отзывов женщин была выявлена острая необходимость признания родов, родовой деятельности и послеродового периода как физиологических и достойных уважения процессов жизнедеятельности человека, вмешательство в которые должно осуществляться только в случае необходимости. Продвижение и (или) повторное внедрение ассистированных вагинальных родов в странах с низкими ресурсами требуют от государств, ответственных лиц и администраторов больниц поддержки квалифицированных медицинских работников, которые в свою очередь могут с уважением относиться к женщинам во время схваток и родов.

## Introduction

Assisted vaginal birth, also known as instrumental or operative vaginal birth, refers to a vaginal birth conducted with the help of an instrument such as forceps or a vacuum extractor.^1^ Common indications for assisted vaginal birth include a prolonged second stage of labour (when the cervix is fully dilated) because of maternal exhaustion or an inability to push effectively, or fetal distress when the head is deeply engaged in the birth canal.^2^^–^^4^ In these situations, and in the absence of action to expedite birth, women and babies are at higher risk of complications such as infection, haemorrhage, fetal compromise, birth asphyxia, meconium aspiration syndrome, lifelong adverse consequences (including obstetric fistula in the women and neurological disabilities in the baby), or even death.^5^^–^^8^

Assisted vaginal birth is not without risks, particularly if conducted inappropriately or by unskilled providers.^9^^,^^10^ Compared with non-instrumental vaginal birth, the use of forceps is associated with an increased risk of perineal trauma, maternal pain and fetal facial injury; the use of a vacuum extractor is associated with a higher risk of cephalohematoma and failure resulting in caesarean section.^1^^,^^11^ However, these comparisons can be misleading, since a woman in need of forceps or a vacuum extractor is experiencing a second-stage complication or emergency that needs to be addressed: a non-instrumental vaginal birth is no longer an option.^9^ Because assisted vaginal birth can reduce maternal and perinatal mortality and morbidity, including obstetric fistula, perinatal hypoxia, infection and postpartum haemorrhage associated with prolonged labour, the World Health Organization (WHO) recognizes it as an integral part of basic emergency obstetric care.^1^^,^^7^^,^^9^^,^^12^^,^^13^ Major and influential obstetric societies worldwide also recognize this practice as an important component of modern childbirth management.^2^^–^^4^^,^^14^^,^^15^

The alternative to assisted vaginal birth for women who have second-stage complications is a caesarean birth. However, compared with a pre-labour or first-stage caesarean birth (i.e. when the cervix is not fully dilated), a caesarean delivery performed during the second stage of labour presents additional risks, including major haemorrhage, infection, extension of the uterine incision and trauma to the baby’s head.^16^ For many women, an assisted vaginal birth can therefore be safer than an emergency second-stage caesarean birth.^7^^,^^9^^,^^13^^,^^17^^,^^18^ The short- and long-term risks associated with a caesarean delivery (at any stage of labour) also need to be considered, including the risks of complications in future pregnancies and on the health of the child later in life.^19^^–^^22^

The cost-effectiveness of these interventions has not been extensively studied. However, in the United States of America, a cost-effectiveness model analysis of neonatal and maternal outcomes of assisted vaginal birth versus caesarean birth was conducted.^23^ This study suggested that assisted vaginal birth was more cost-effective, with analyses indicating higher quality-adjusted life years of the mother and neonate as well as reduced costs.

## Declining practice 

Despite current evidence reporting the greater risks and higher costs of caesarean birth compared with assisted vaginal birth, the number of caesarean births has increased while the practice of assisted vaginal birth has declined, particularly in low- and middle-income countries.^24^^–^^26^ In the last three decades, the proportion of all births by caesarean section has increased from a global average of about 6% in 1990 to 21% in 2018.^25^^,^^27^ Concurrently, the use of assisted vaginal birth is mostly limited to high-income countries in which 4%–15% of all births are assisted with forceps or vacuum extractor.^28^^–^^30^ Despite the fact that caesarean births entail greater risks in low- and middle-income countries – a result of the poorer quality of services and reduced access to comprehensive obstetric care in the case of complications – assisted vaginal births comprise less than 1% of all births in such settings.^5^^,^^26^^,^^31^^,^^32^ Increased access to the practice of assisted vaginal birth can support efforts towards reducing the use of second-stage caesarean birth and its associated short- and long-term risks.

Multiple factors contribute to the underuse of assisted vaginal birth globally: the practice requires clinical judgement, expertise and skills, but there exists a lack of adequately trained health-care providers, not only to conduct the procedure but also to identify which labouring women could benefit.^26^^,^^33^^,^^34^ Building expertise takes time, practice, supervision and support,^33^^–^^35^ while strategies to optimize training effectiveness for obstetric emergencies remain elusive.^35^^–^^37^ The lack of functioning, reliable and affordable equipment at the point-of-care is also a limiting factor to sustainability,^1^^,^^26^^,^^34^ as is the lack of or suboptimal pain relief.^38^ Concerns about safety and fear of complications associated with the practice act as deterrents among both women and health-care providers, as well as fear of litigation in health-care providers in the case of adverse events.^38^^–^^40^ Because it is widely perceived as safer and more modern than vaginal birth, and a way to guarantee the best outcome for mother and child, caesarean birth is becoming a social norm among women and their relatives.^40^^,^^41^

## Three-step process

WHO recognizes the crucial role of assisted vaginal birth in improving maternal and perinatal outcomes, but also acknowledges the need to conduct research to identify and address barriers to its implementation. WHO therefore led a three-step process to foster thinking and catalyse research on how to optimize the use of assisted vaginal birth, especially in low- and middle-income countries: (i) evidence syntheses; (ii) technical consultation with experts; and (iii) gathering of feedback and views from women’s representatives. We combined the results and outcomes from all three steps to identify the areas in which research, political engagement and support, and resources are urgently required. 

### Evidence syntheses

First, we conducted one quantitative and two mixed-methods systematic reviews of the global literature describing research on barriers to, and factors facilitating the use of, assisted vaginal birth. Our quantitative review synthesized evidence from 16 studies (10 based in low- and middle-income countries) that implemented interventions to increase or reintroduce the use of assisted vaginal births, including didactic sessions, simulation, hands-on training, guideline production and audit/feedback.^37^ Our mixed-methods reviews aimed to improve our understanding of (i) experiences and facilitators of, as well as barriers to, assisted vaginal birth (42 studies);^38^ and (ii) competencies and expertise required, as well as barriers to and facilitators of, such competencies and expertise, from the point of view of health-care professionals (27 studies).^34^

### Consultation with technical experts

This synthesis of evidence informed a consultation with 37 technical experts, including health- care providers, researchers, policy-makers and public health experts selected for their wide range of experience, perspectives and geographical locations. A research agenda required for the safe reintroduction of, or increased access to and use of, assisted vaginal birth, especially in low- and middle-income countries, was tentatively identified by participants of the consultation. 

### Women’s views

Following the experts’ initial identification of a research agenda, we ascertained the perceived importance and relevance of this agenda at four workshops to which representatives of women’s and advocacy groups from 27 different countries were invited. To accommodate the range of languages spoken and geographical locations, the workshops were conducted online (each lasting 1.5–2 hours) in English (two), French (one) and Spanish (one) during April–May 2022. Each workshop was conducted by two independent professional facilitators contracted to WHO. Around a week before each workshop, participants were sent the tentatively identified research agenda so that they could begin to consider and construct their views on each research topic. The facilitators led discussions during the workshops and noted the opinions expressed; the facilitators then prepared reports detailing the women’s views on the importance and relevance of the topics on the identified research agenda.

## Research agenda

Earlier in 2023, WHO published a technical brief describing the results of this process; please see this technical brief for a tabulated form of the following.^42^ We categorized the topics within the identified research agenda as: (i) women’s perception of assisted vaginal birth; (ii) training of health-care providers and clinical aspects; and (iii) implementation and sustainability of the practice. The key messages and implications of the process and outcomes in each of the three categories are discussed below, and include the views and perspectives developed by both the technical consultants and women’s representatives.

### Perception

Accessing reliable information and debunking misconceptions about assisted vaginal birth may be a challenge in contemporary societies. Although most women have basic information about a caesarean birth, many are less familiar with assisted vaginal birth. The views of those who are aware of the practice may have been affected by catastrophic but rare outcomes, undermining evidence-based information and reliable statistics. 

WHO therefore emphasizes the need for research to understand how assisted vaginal birth is perceived by women and communities, and also how to provide appropriate, reliable and unbiased information about the practice. Determining the most effective ways to communicate information to women and their communities across different settings (e.g. low levels of literacy, poor internet availability, marginalized or minority populations) is crucial. The provision of decision-making tools for women as well as the role of various communication channels (e.g. social media) should both be explored.

Women emphasized the importance of recognizing childbirth as a physiological process, as opposed to an illness. Recurrent concerns about assisted vaginal birth reported at the workshops were the safety of the practice, and the consequences of adverse events on both the newborn and mother. Women were greatly troubled by an apparent lack of follow-up and support (e.g. expressed in concerns about overburdened health systems and a lack of infrastructure and equipment) in the case of complications. Finally, the misuse of informed consent and its consequences (e.g. fear, disempowerment and abuse) was identified as a source of distress for women. 

### Training and clinical aspects

The importance of well-trained, skilled, knowledgeable and competent health-care providers cannot be overemphasized. Research is needed to identify the most effective ways to educate and train health-care providers to not only perform assisted vaginal birth, but also to recognize the indications and conditions for its safe use, the contraindications and how to manage any complications. The latter is particularly crucial since fear of complications is a documented deterrent for health-care providers considering offering assisted vaginal birth. In addition to improving the clinical skills of providers, training should include other skills such as effective communication, situation awareness, shared decision-making, informed consent, and how to respectfully guide women and their birth companions and/or family through the birth experience. 

Subsequently, it is essential to develop comprehensive guidelines and training packages that can be used and adapted at country level. As country-level opinion leaders, national professional associations must play an active role in preparing, updating and endorsing management guidelines for labour, childbirth and assisted vaginal birth that are both evidence-based and comprehensive. Professional associations must also support effective training and regulation that is conducive to high-quality assisted vaginal birth as an integral part of obstetric care.

To be able to address recurrent concerns about the safety of the practice, studies comparing the effect of assisted vaginal birth with second-stage caesarean birth on both the short- and long-term maternal (e.g. the effect on the pelvic floor) and newborn outcomes are needed. In terms of clinical practice, research is needed on the optimal analgesia methods for assisted vaginal birth across different settings, and on the safety, feasibility and effectiveness of new technologies to perform the practice such as the OdonAssist device.^1^^,^^43^^,^^44^ Development of technologies that are simple, user-friendly and reusable may have the most impact.

The labour and birth environment (e.g. maternity structures, resources, support and organizational ethos) has the potential to represent either an insurmountable barrier or an important ally in access to high-quality care for pregnant women. It is therefore important to investigate (i) the factors that incentivize caesarean births and foster inequalities in access to assisted vaginal birth; and (ii) the changes required in the culture of obstetric health-care institutions and systems to prevent disrespect, abuse or overmedicalization.

### Implementation and sustainability

To implement and sustain the practice of assisted vaginal birth, particularly in settings where second-stage caesarean birth may be a riskier option, research is needed to assess maternity organizational models for effective access. Because midwives are the main providers of health care in rural and remote areas of many low- and middle-income countries, assessing the safety, feasibility and effectiveness of midwife-led models of care; exploring the role of midwives, as well as investigating how to train and empower them to undertake assisted vaginal birth safely and effectively, is fundamental. Regardless of the organizational model, ensuring timely access to emergency caesarean section if assisted vaginal birth fails is also crucial. Health-care providers need a supportive work environment, including relevant training, mentoring by more experienced colleagues, and close supervision until an appropriate level of confidence and competence is reached.

Other questions that need to be answered include how to manage political and financial barriers, including the structure of the insurance system. Change will be difficult as long as a caesarean birth is more profitable for the system.^45^ It is important to ensure that funding is available to acquire and maintain essential equipment, and to determine which interventions other than education (e.g. audit and feedback, opinion leaders) could be effective in changing obstetric practices that are acceptable to both women and health-care providers. Studies demonstrating the cost–effectiveness of the increasing use of assisted vaginal birth are crucial for the engagement of governments, policy-makers and professional associations.

In current obstetrics, the challenge of increasing the use of assisted vaginal birth is complex because of the interconnected multiple players (e.g. women, health-care providers, policy-makers and professional associations); accepted cultural norms; the dynamic health-care environment; and the numerous behavioural factors (e.g. fear of pain, a poor outcome, or disrespect during labour) that influence decision-making for mode of birth.^45^ Such complexity requires operational implementation research, using a systems-thinking approach with its toolbox of motivation changers (e.g. accountability, peer pressure, champions, emerging leadership).^46^ The systematic use of behavioural science is also warranted to address all barriers and concerns while harnessing the factors facilitating appropriate use.^47^^,^^48^

## Conclusion

Access to assisted vaginal birth is an integral part of emergency obstetric care; we have therefore identified a research agenda to counter the significant decline in its use. Women’s representatives generally agreed with the research agenda initially identified by the technical experts, but also stressed the importance of engaging women and health-care professionals at all stages of this research. Women also emphasized the urgent need to recognize labour, childbirth and postpartum experiences as inherently physiological and dignified human processes, in which interventions should only be implemented if and when needed. The promotion and/or reintroduction of assisted vaginal birth in low-resource settings requires governments, policy-makers and hospital administrators to support skilled health-care providers who can, in turn, respectfully support women in labour and childbirth. Shared and informed decision-making and constructive communication are considered crucial for reducing apprehension and building trust between women and health-care providers.
